# ASO Author Reflections: Is There Any Difference Among Various Gleason Scores Classified as Grade Group 4 Prostate Cancer?

**DOI:** 10.1245/s10434-021-10335-0

**Published:** 2021-06-21

**Authors:** Keiichiro Mori, Vidit Sharma, Shin Egawa, Derya Tilki, Stephen A. Boorjian, Shahrokh F. Shariat

**Affiliations:** 1 Department of Urology, Medical University of Vienna, Währinger Gürtel 18-20, 1090 Vienna, Austria; 2 Department of Urology, The Jikei University School of Medicine, Tokyo, Japan; 3 Department of Urology, Mayo Clinic, Rochester, MN USA; 4 Department Of Urology, VA Health Services Research and Development Fellowship, University of California, Los Angeles, CA USA; 5 Martini-Klinik Prostate Cancer Center, University Hospital Hamburg-Eppendorf, Hamburg, Germany; 6 Department of Urology, University Hospital Hamburg-Eppendorf, Hamburg, Germany; 7 Institute for Urology and Reproductive Health, Sechenov University, Moscow, Russia; 8 Division of Urology, Department of Special Surgery, The University of Jordan, Amman, Jordan; 9 Department of Urology, Weill Cornell Medical College, New York, NY USA; 10 Department of Urology, University of Texas Southwestern, Dallas, TX USA; 11 Karl Landsteiner Institute of Urology and Andrology, Vienna, Austria; 12 Department of Urology, Second Faculty of Medicine, Charles University, Prague, Czech Republic

## Past

Currently, grade group (GG) 4 prostate cancer (PC) is equivalent to Gleason score (GS) 8 PC, which consists of GS 4+4, 3+5, and 5+3. Grade group 4 still is considered a homogeneous entity with regard to its associated prognosis and treatment. However, some reports have raised questions as to whether GS 8 or GG 4 is a heterogeneous entity in terms of prognosis, and hence whether there is merit in reclassifying GG 4 into separate GGs.^1^ The authors have shown prognostic differences in patients who have PC in GG 4 treated with radical prostatectomy (RP) based on different Gleason scores for RP specimens, suggesting that considerable heterogeneity exists within GG 4 regarding oncologic and surgical pathologic outcomes.^2^ However, discrepancy exists between the biopsy and RP GS, with the two specimens matching exactly in approximately 50% of cases.^3^ Thus, it remains unclear whether our findings on RP specimens also hold true for biopsy specimens.

## Present

This study was conducted to investigate the prognostic differences between GS 3+5, GS 4+4, and GS 5+3 in biopsy specimens from patients with PC classified as GG 4 based on the association with oncologic and surgical pathologic outcomes.^4^ In this multicenter retrospective study, 1791 patients (GS 3+5: 190; GS 4+4: 1557; and GS 5+3: 44) with biopsy GG 4 were included. During a median follow-up period of 75 months, 750 patients experienced biochemical recurrence (BCR), 146 died of any cause, and 57 died of PC. The results indicate that GS 5+3 was associated with significantly higher rates of GS upgrading in RP specimens than GS 3+5 or GS 4+4. In contrast, no association was found between GS and lymph node metastases, non-organ-confined disease, positive surgical margin, or extraprostatic extension disease. Moreover, GS was not associated with overall survival or cancer-specific survival, but was associated with BCR-free survival (*P* = 0.03).

## Future

In this study, the patients with biopsy GG 4 exhibited some limited but clinically significant heterogeneity. Indeed, significant differences were seen in association with GS upgrading, downgrading, and BCR. The absence of central reviews involving expert pathologists is a limitation of this study given that earlier studies lacking central reviews indeed were associated with high proportions of patients with GS 3+5 and GS 5+3 and that a high percentage of GS 3+5 and GS 5+3 has been re-categorized upon expert review.^5^ Well-designed prospective studies with prolonged follow-up evaluation and central pathologic review are warranted to validate the differential prognostic and biologic impact of the different GS within GG 4 in the clinical setting.
